# Climate and density influence annual survival and movement in a migratory songbird

**DOI:** 10.1002/ece3.1854

**Published:** 2015-12-01

**Authors:** Ann E. McKellar, Matthew W. Reudink, Peter P. Marra, Laurene M. Ratcliffe, Scott Wilson

**Affiliations:** ^1^ Canadian Wildlife Service, Environment Canada115 Perimeter RoadSaskatoonSaskatchewanS7N 0X4Canada; ^2^ Department of Biological SciencesThompson Rivers UniversityKamloopsBritish ColumbiaV2C 0C8Canada; ^3^ Migratory Bird CenterSmithsonian Conservation Biology InstituteWashingtonDistrict of Columbia20013‐7012; ^4^ Department of BiologyQueen's UniversityKingstonOntarioK7L 3N6Canada; ^5^ Wildlife Research Division, Environment CanadaNational Wildlife Research Centre1125 Colonel by DriveOttawaOntarioK1A 0H3Canada

**Keywords:** American redstart, annual survival, breeding dispersal, density, El Niño Southern Oscillation, multistate mark–recapture, Normalized Difference Vegetation Index, *Setophaga ruticilla*

## Abstract

Assessing the drivers of survival across the annual cycle is important for understanding when and how population limitation occurs in migratory animals. Density‐dependent population regulation can occur during breeding and nonbreeding periods, and large‐scale climate cycles can also affect survival throughout the annual cycle via their effects on local weather and vegetation productivity. Most studies of survival use mark–recapture techniques to estimate apparent survival, but true survival rates remain obscured due to unknown rates of permanent emigration. This is especially problematic when assessing annual survival of migratory birds, whose movement between breeding attempts, or breeding dispersal, can be substantial. We used a multistate approach to examine drivers of annual survival and one component of breeding dispersal (habitat‐specific movements) in a population of American redstarts (*Setophaga ruticilla*) over 11 years in two adjacent habitat types. Annual survival displayed a curvilinear relation to the Southern Oscillation Index, with lower survival during La Niña and El Niño conditions. Although redstart density had no impact on survival, habitat‐specific density influenced local movements between habitat types, with redstarts being less likely to disperse from their previous year's breeding habitat as density within that habitat increased. This finding was strongest in males and may be explained by conspecific attraction influencing settlement decisions. Survival was lowest in young males, but movement was highest in this group, indicating that apparent survival rates were likely biased low due to permanent emigration. Our findings demonstrate the utility of examining breeding dispersal in mark–recapture studies and complement recent work using spatially explicit models of dispersal probability to obtain greater accuracy in survival estimates.

## Introduction

Given conservation concerns over recent and ongoing declines in many migratory bird populations (Sauer and Link 2011), a key research need is to determine the causes of population limitation and when they occur (Faaborg et al. 2010). Migratory birds are vulnerable to stressors during different times of year and across often vastly different geographic regions. Thus, limitation can be caused by conditions experienced during breeding, nonbreeding, or migratory phases of the annual cycle (Newton 2004), and events experienced during one phase can carry over to influence performance during subsequent phases (Marra et al. 1998; Reudink et al. 2009). In particular, the importance of weather as a driver of bird demographic rates has long been recognized (Lack 1954; Newton 1998).

Local weather patterns influence primary productivity (Lieth 1973) and insect abundance (Janzen and Schoener 1968), which in turn can affect the body condition of birds (Brown and Sherry 2006; Studds and Marra 2007) and ultimately their survival (Robinson et al. 2007). Local weather conditions are driven in large part by global climate cycles (Rogers 1988; Holmgren et al. 2001), which are often better predictors of ecological processes than are small‐scale weather indices (Hallett et al. 2004). In the Caribbean region where many Neotropical migrants overwinter, high values of the Southern Oscillation Index (SOI) are indicative of La Niña conditions and generally result in wetter winters, whereas low values are indicative of El Niño conditions and are associated with milder and drier winters (Ropelewski and Halpert 1987). Previous work on Neotropical migrants in Jamaica found lower survival following El Niño winters (Sillett et al. 2000). The NDVI (Normalized Difference Vegetation Index) has also been used to describe weather conditions experienced by migratory birds. Higher NDVI values are indicative of greater primary production and are generally associated with wetter conditions that are favorable to birds across breeding (Pillar et al. 2015) and nonbreeding periods (Saino et al. 2004).

Intrinsic factors such as population density can also influence survival rates, potentially contributing to negative density‐dependent population regulation (Newton 1998). Multiple mechanisms, such as site dependence or crowding, can contribute to such density dependence (Rodenhouse et al. 1997), and these mechanisms can act on fecundity, survival, or both (Sutherland 1996). The relative strengths of stochastic climatic effects versus density dependence in contributing to demographic variation and population dynamics remain a key focus of research in population ecology (Leirs et al. 1997; Grosbois et al. 2008).

To assess the effects of climate, density, and other factors on bird survival, researchers generally rely on mark–recapture studies of color‐banded birds. However, most studies estimate apparent survival, which is the product of both true mortality and fidelity to the study area (Lebreton et al. 1992). For migratory birds, patterns of annual survival on the breeding grounds may be obscured if there is temporal variation in breeding dispersal (Marshall et al. 2004), defined as the movement of adults between breeding attempts. Existing methods that quantify rates of emigration generally rely on live recapture and dead recovery data that allow for the separation of true survival and permanent emigration (Burnham 1993), but these data are rarely available for nonharvested species. More recent spatially explicit mark–recapture approaches make use of the locations of individual encounters to model dispersal probabilities (Gilroy et al. 2012; Ergon and Gardner 2014; Schaub and Royle 2014). However, these methods require the precise locations of capture for each individual in each year and specific assumptions (e.g., individuals have unique centers of activity where capture is most likely to occur; movement direction is unrelated to location). An alternative approach which may be more feasible with limited data is to quantify dispersal rates within a study area to infer patterns of dispersal more generally (Lebreton et al. 2009; Schaub and von Hirschheydt 2009).

As with survival, dispersal can be influenced by climate and population density. Natal dispersal in American redstarts (*Setophaga ruticilla*) is influenced by nonbreeding habitat quality (Studds et al. 2008), and both natal and breeding dispersal may be related to annual variation in breeding season phenology (Rushing et al. 2015a). Greater population densities can lead to higher dispersal if competition and resource depletion encourage individuals to disperse (Paradis et al. 1998), or lower dispersal through mechanisms such as conspecific attraction (Fretwell and Lucas 1970; Muller et al. 1997). Public information can also influence patterns of breeding dispersal based on a site's reproductive output (Doligez et al. 2002; Parejo et al. 2007).

The relative influence of factors such as climate and density might vary among sex and age classes. In Eurasian spoonbills (*Platalea leucorodia leucorodia*), a positive effect of density on annual survival was apparent at low population sizes in younger but not in older birds, whereas younger birds showed a stronger decrease in survival with increasing population size (Lok et al. 2013). In American redstarts, females and young birds are often excluded from high‐quality nonbreeding habitat by dominant older males (Marra 2000) and thus may suffer greater effects on their annual survival in years of unfavorable nonbreeding season weather (Studds and Marra 2007). Unfortunately, such age‐ and sex‐specific interaction effects with annual covariates are often difficult to discern in mark–recapture studies due to large sample size requirements.

In this study, we used multistate mark–recapture models to examine potential drivers of age‐ and sex‐specific annual survival in a population of American redstarts over 11 years. We further made use of the existence of two spatially separate deciduous habitat types within our study area that differed in age structure and landscape continuity to examine factors influencing one component of breeding dispersal: habitat‐specific movements across years. We first asked how apparent survival and movement varied among age and sex classes. We then tested the effects of potential drivers of annual variation in survival and movement. For the second objective, we made the following predictions: (1) more favorable nonbreeding season weather (high SOI, high NDVI) would be associated with higher survival, with less of an influence of local breeding season weather (Hallett et al. 2004) and that (1a) these effects would be strongest in females and young birds (Studds and Marra 2007); (2) survival would be lower following years of higher density (Newton 1998); (3) movements would be lower with increasing habitat‐specific density the current year, due to conspecific attraction (Hahn and Silverman 2006), and following higher reproductive success the previous year (Doligez et al. 2002).

## Materials and Methods

### Field methods

American redstarts are small (7–8 g), Nearctic–Neotropical migrant songbirds that breed across the northern United States and Canada and spend the boreal winter across Mexico, the Caribbean, and parts of Central and South America (Sherry and Holmes 1997). Based on feather stable isotope analysis, redstarts overwintering in the Caribbean appear to breed primarily in the northeastern United States and southeastern Canada, including our study site (Norris et al. 2006).

Field work for this project was conducted from May–July 2001–2011 at the QUBS (Queen's University Biological Station), Chaffey's Lock, Ontario, Canada (44°34′ N, 76°19′ W). The study area includes two major habitat types: “undisturbed forest” (hereafter forest; 75 ha) and “campground” (25 ha), divided by a two‐lane country road. Both habitats are mixed‐deciduous forest, dominated by sugar maple (*Acer saccharum*) and Eastern hop hornbeam (*Ostrya virginiana*). Although the habitats are adjacent, they differ markedly in terms of landscape continuity, edge habitat, and human presence. The forest is largely contiguous, disrupted only by walking trails and several small wetlands. In contrast, the campground to the south of the country road is composed of a mix of cottages, recreational vehicle campsites, and tent campsites connected by a network of roads and trails. Due to these light disturbances, the campground contains a greater proportion of early‐successional components than the mature forest. In addition, the campground area borders on Lake Opinicon with ~1 km of shoreline on its south boundary.

Male redstarts arrive on the breeding grounds several days prior to females and begin singing immediately upon arrival to demarcate territory boundaries and attract females. During the arrival period (May 1–31), we surveyed the entire study area each day from 0600 to 1200, detecting males through singing, mapping territory boundaries, and identifying returning individuals by the presence of leg bands. Birds on each territory were followed for at least 20–30 min/day and locations where birds were observed were noted on paper maps with the help of landmarks, trails, and camp numbers in the campground, and a grid of flagged trees in the forest. For birds mapped over multiple years, we determined approximate movement distances from 1 year to the next based on the mapped territories. Due to the inexact nature of the mapping, we grouped movement distances into 100 m categories (<100, 100–200, 200–300 m, etc.). We recorded pairing date and the location of the nest. Once nest‐building commenced, we recorded the number of eggs laid, hatching success, and fledging success (see Reudink et al. 2009).

Males, and occasionally females, were captured shortly after arrival, using mist nets with song playback and a decoy to simulate a territorial intrusion. Females were primarily captured during feeding trips to the nest or using fledgling distress calls to lure them into the net. Upon capture, all individuals were given a single USGS aluminum band and 2–3 color bands for individual identification. Male American redstarts exhibit delayed plumage maturation and were aged based on plumage coloration; after second year (ASY), males are black with orange patches on the tails, wings, and flanks, whereas second year (SY) males exhibit the same patterning but resemble females with gray and yellow plumage. Females were identified by plumage coloration and the presence of a brood patch and aged based on the degree of wear and coloring of the rectrices and molt limits, following Pyle (1997).

We considered annual territory density for each habitat type as the total number of pairs and unmated territorial males each year (Table 1; see McKellar et al. 2014). We calculated annual mean fledging success for each habitat type by dividing the total number of offspring successfully fledged by the number of females monitored throughout the entire season each year. We considered a habitat‐specific movement event to occur when an individual that previously held a territory in one habitat type was found to hold a territory in the other habitat type in a subsequent year.

**Table 1:** Annual territory density (number of territories), mean fledging success (fledglings per female), and habitat‐specific movement for American redstarts in forest (F) or campground (C) type habitats. Movement events are indicated for the year in which movement into the new habitat type occurred

Year	F. territories	C. territories	F. fledging success	C. fledging success	Movement F – C	Movement C – F
2001	9	19	2.44	3.40		
2002	15	25	1.08	2.81		
2003	21	23	1.88	1.44	3	
2004	26	17	2.13	2.20	1	
2005	34	23	0.86	1.00		
2006	33	8	1.77	0.60	2	
2007	30	17	2.00	0.67	1	3
2008	32	15	1.64	1.10	2	
2009	22	17	2.93	1.80		1
2010	23	19	1.46	2.17	1	1
2011	18	22	0.08	1.00		

### Climate and vegetation indices

We obtained mean monthly values for the SOI from the NOAA (National Oceanic and Atmospheric Administration) Climate Prediction Centre (http://www.cpc.ncep.noaa.gov/data/indices/). We used the mean SOI during the dry season from December through March as a proxy to reflect the conditions experienced by American redstarts during the nonbreeding period. Weather during this time period in the Caribbean is associated with food availability (Studds and Marra 2007), departure dates from the nonbreeding grounds (Studds and Marra 2011), arrival to the breeding grounds (McKellar et al. 2013), and subsequent breeding season abundance (Wilson et al. 2011).

We obtained NDVI values from the NASA Land Processes Distributed Active Archive Center (LP DAAC; https://lpdaac.usgs.gov/data_access). The NDVI is calculated as (NIR − Red)/(NIR + Red) with NIR (near‐infrared) and Red being equal to the amount of near‐infrared and red light, respectively, reflected by a surface and recorded by remote sensing (Jensen 2007). The NDVI is positively correlated with measures of plant productivity such as leaf area index and canopy extent (Hicke et al. 2002; Wang et al. 2005). Data were provided as monthly 1 km resolution products from the MODIS (Moderate Resolution Imaging Spectroradiometer) Terra dataset. From these, we calculated annual mean NDVI from December through March for islands in the Greater Antilles. Due to high correlations between NDVI from Cuba and Jamaica (*r* = 0.70), and from Hispaniola and Puerto Rico (*r* = 0.69), we computed area‐weighted means for the two regions and used separate western (Cuba/Jamaica) and eastern (Hispaniola/Puerto Rico) NDVI covariates in our models.

Local breeding season temperature and rainfall data were obtained from a weather station at QUBS. The station provided mean daily temperature and daily total rainfall, and we used these to calculate mean temperature and total rainfall for the breeding season (June–July) each year.

### Multistate capture–recapture model

To examine survival and breeding dispersal, we used the spatial configuration of the habitat types in a multistate mark–recapture approach (Nichols et al. 1994; Lebreton et al. 2009), which provides estimation of state‐specific probabilities of apparent survival and recapture as well as the transition probabilities between states. While a multistate approach cannot separate true survival and permanent emigration, it can provide an indication of breeding dispersal between habitat types and the extent to which it varies with sex and age and in relation to our predictions. Multistate analyses were conducted in program MARK (White et al. 2006).

Each individual's encounter history contained an “F” or a “C” as the two states dependent on whether the individual held a territory in forest (F) or campground (C), respectively, in year *t−*1. As an example, we illustrate the associated probabilities for an individual in state *F* at time *t−*1 (i.e., establishes in forest habitat). This individual will either survive (*ϕ*_F_) or die (1 *− ϕ*_F_) during the interval *t−*1 to *t* as a partial function of having been established in forest at time *t−*1. If the individual survives the interval and returns to the study area, it can remain in state *F* at time *t* with probability *ψ*_FF_ (i.e., establishes again in forest) or move to state C with probability *ψ*_FC_ = 1 *−* *ψ*_FF_ (i.e., establishes in campground). Thus, as defined here, the individual survives the interval prior to the movement event. This is a reasonable assumption for Neotropical migrants as we expect they return to their former territory during the prebreeding period in year *t* and subsequently disperse as opposed to dispersing between habitats prior to fall migration in year *t−*1. Regardless, this assumption pertains to the influence of an individual's state in year *t−*1 on survival from *t−*1 to *t*. Even if they prospect in the new habitat during the postbreeding period, we expect that survival is influenced by their habitat choice in year *t−*1 and our models were structured to represent survival followed by dispersal. Conditional on the individual surviving the interval and returning to the study area, it can then be recaptured at time *t* with probability *p*_F_ in forest or *p*_C_ in campground. An individual that has permanently emigrated from the study area cannot be recaptured and thus multistate models as used here can only provide a minimum estimate of breeding dispersal.

### Estimating apparent survival, recapture, and movement

Model support was evaluated using AIC (Akaike's information criterion, Burnham and Anderson 2002), and model fit was examined using the median $\hat{c}$ test on the most general model. Our model set did not contain a fully time‐dependent model, and therefore, a goodness‐of‐fit test was only possible with median $\hat{c}$ (Cooch and White 2013). If there was lack of fit, we used *ĉ* to adjust standard errors, and the ΔQAIC values and Akaike weights (*w*_*i*_) were used to infer support for each of the candidate models.

Our analyses included two objectives: (1) estimation of how survival and movement rates varied between sex and age classes, and (2) tests of predictions on the mechanisms that influenced survival and movement. Our modeling approach began with objective 1, and we used a model that included additive effects of sex and age on survival and state transition (i.e., movement) probabilities to identify the recapture structure with the greatest support. To examine recapture, we included sex, age, and habitat as additive effects along with reduced models that excluded age and/or habitat. Due to larger sample sizes of males (*n* = 445) than females (*n* = 211), we always included sex in the models. We also included interactions of sex by habitat and sex by age on recapture probability. After evaluating support for recapture, we considered additional survival and transition model structures with habitat, interactions of sex and age, as well as reduced models without age effects. We obtained the real parameter estimates for survival and movement by age and sex using model averaging across the candidate set.

For objective 2, we used the most well‐supported model from objective 1 that also allowed us to test all predictions. For apparent survival from year *t−*1 to *t*, we considered SOI and NDVI in year *t−*1(December) to year *t* (January–March), breeding season weather in year *t−*1 (June–July temperature and precipitation), and habitat‐specific density in year *t−*1. The three nonbreeding season variables were included individually because the El Niño Southern Oscillation affects rainfall in the Caribbean (Ropelewski and Halpert 1987) and may influence plant productivity at larger scales measured by the NDVI (Poveda et al. 2001). For SOI and the two breeding season weather variables, we considered both linear and quadratic relationships because it is possible that survival is lower at the extremes (e.g., El Niño and La Niña events). All annual variables were standardized prior to analysis with mean 0 and standard deviation 1. Because models included interactions, we did not model average the beta coefficients and instead report coefficients for annual covariate effects from the top model containing the respective variable.

To evaluate the influence of annual covariates, we added each covariate individually to the best model without covariates and evaluated support in terms of change in QAICc (including interactions of these variables with age and sex). Covariates whose inclusion led to a drop in QAICc were then included together in the top model. We also examined support for reduced versions of this model because correlated variables may explain the same variance in the response. Finally, we added each of the previously excluded annual covariates back into the top model and evaluated their support again because it is possible that influential variables must be in the model to identify weaker effects of other covariates. This latter step will result in models that differ in only one parameter and are within 2 QAICc units of the top models but appear competitive only because of the variables in the top model. Therefore, if the addition of these variables did not lead to a reduction in QAICc, the model was removed (see Devries et al. 2008).

For habitat‐specific movement, we included the effects of (1) mean fledging success in an individual's habitat in year *t−*1 and (2) density in year *t* of the habitat in which an individual occurred in year *t−*1. Due to low sample sizes, we only tested these variables as a uniform response across sex and age classes, and as a uniform response among age classes in males only.

## Results

Between 2001 and 2010, we marked 211 females (117 ASY, 94 SY) and 445 males (261 ASY, 184 SY). Of these, 127 individuals were observed in at least one year subsequent to marking and several were observed in two or more years (mean ± SD = 2.4 ± 0.7 years). These 127 individuals included 20 ASY females, 20 SY females, 65 ASY males and 22 SY males. Total movements between habitats were few; of 178 cases where an individual had a known habitat in year *t−*1 and was observed in year *t*, 163 returned to the same habitat while 15 switched habitats (three females, 12 males). The majority of habitat‐specific movements were from forest to campground (Table 1).

**Figure 1:**
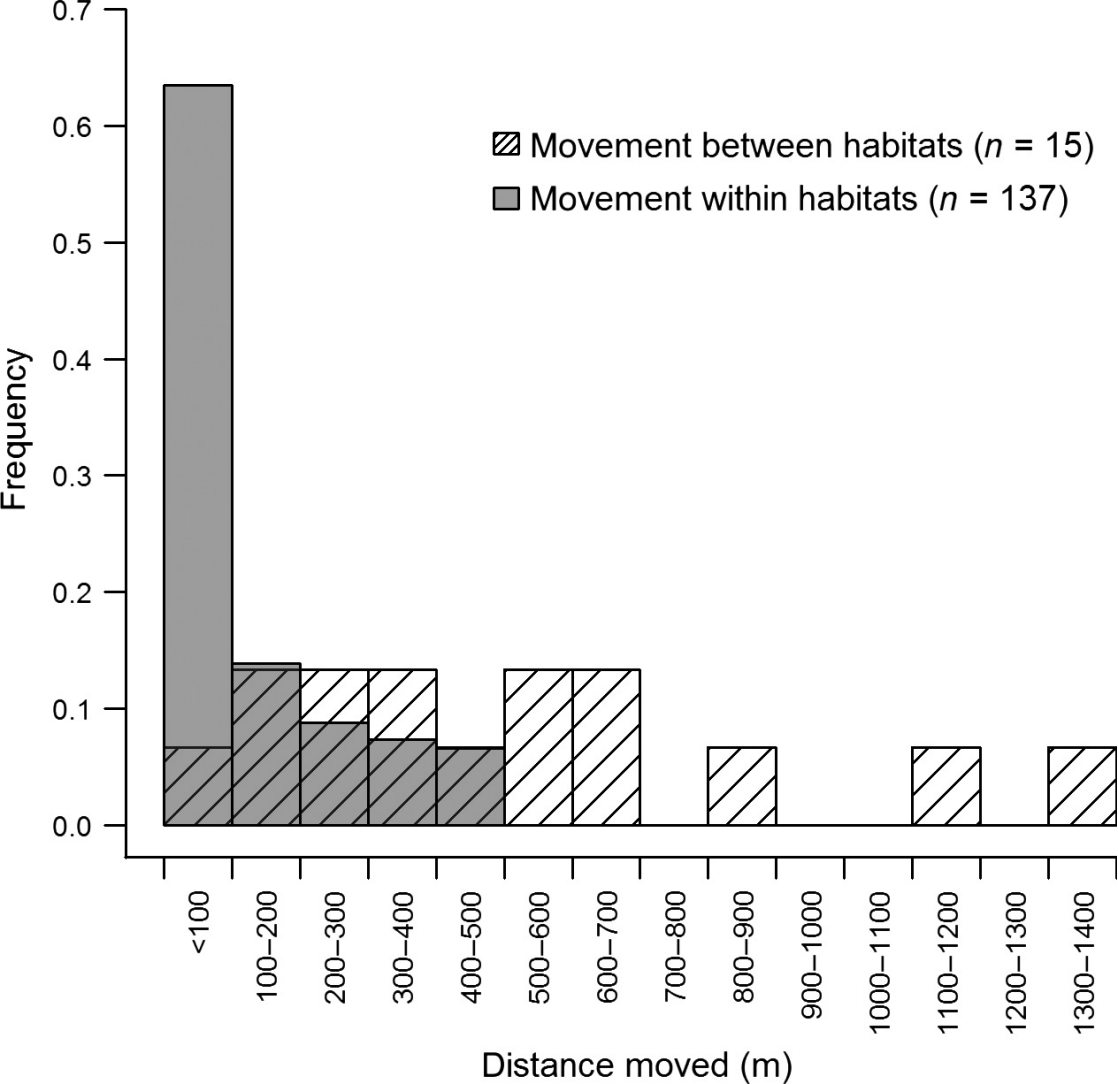
Frequency histogram of distances moved for American redstarts that moved between habitat types (*n* = 15) or remained within the same habitat type (*n* = 137) across years.

Of the 178 cases when an individual returned in a subsequent year, we were able to determine the approximate distance moved for 152 cases, including all 15 movements between habitat types and 137 movements within a habitat type (Fig. 1). For the remaining cases, mapping details were not sufficient to categorize the movement distances. For movements within habitat types, birds were often found to return to the same or very nearby territories. In contrast, movements that occurred between habitat types often featured longer distances. At the same time, it was possible for individuals to switch between habitat types and yet move shorter distances than individuals that remained within the same habitat type (Fig. 1).

### Objective 1: survival, recapture, and movement probabilities

The estimate of ĉ for the most general model was 1.18, indicating a reasonable fit of the model to the data. We fit 56 models in total, with 16 for objective 1 and 40 for objective 2. The recapture model with the greatest support included habitat and sex; recapture rates were higher for males and in campground (male campground: 0.91 ± 0.04, male forest: 0.70 ± 0.10, female campground: 0.53 ± 0.13, female forest: 0.21 ± 0.07, Table 2). Model‐averaged estimates of apparent survival showed similar values for the two age classes among females but a strong difference among males, with SY birds displaying far lower apparent survival compared to ASYs (Table 3). Apparent survival of both male age classes was lower than female apparent survival. There was little support for an effect of habitat on apparent survival.

**Table 2:** Model selection results of factors influencing apparent survival (*ϕ*), recapture (*p*), and movement rates (*ψ*) in American redstarts. The change in Akaike's information criterion based on quasi‐likelihood with small sample correction bias (ΔQAICc), QAICc weights (*w*_*i*_), number of model parameters (*k*), and model deviance (Qdev) are shown. We examined 56 candidate models and present the top 10 with Σ*w*_*i*_ > 0.99 as well as the top model containing variation in *ϕ*,* p* and *ψ* by sex, age and habitat prior to adding annual covariates (bolded)

Model^a^	ΔQAICc	*w*_*i*_	*k*	Qdev
*ϕ*_sex*age+SOIsq+rain_ p_sex*age+habitat_ *ψ*_sex+age+ density (*t−*1).male*_	0	0.35	16	266.84
*ϕ*_sex*age+SOIsq*age+rain_ p_sex*age+habitat_ *ψ*_sex+age+ density (*t−*1).male_	0.39	0.28	18	263.06
*ϕ*_sex*age+SOIsq+rain_ p_sex*age+habitat_ *ψ*_sex+age_	1.07	0.20	15	266.99
*ϕ*_sex*age+SOIsq+rain_ p_sex*age+habitat_ *ψ*_sex+age+rep.succ_	2.19	0.12	16	269.03
*ϕ*_sex*age+SOIsq*sex+rain_ p_sex*age+habitat_ *ψ*_sex+age_	5.02	0.03	17	269.78
*ϕ*_sex*age+SOI*age+rain_ p_sex*age+habitat_ *ψ*_sex+age_	7.92	0.01	15	276.84
*ϕ*_sex*age+SOIsq*age*sex+rain_ p_sex*age+habitat_ *ψ*_sex+age_	8.06	0.01	21	264.43
*ϕ*_sex*age+SOIsq_ p_sex*age+habitat_ *ψ*_sex+age_	9.27	0.00	16	276.11
*ϕ*_sex*age+SOI+rain_ p_sex*age+habitat_ *ψ*_sex+age_	10.33	0.00	14	281.32
*ϕ*_sex*age+rain_ p_sex*age+habitat_ *ψ*_sex+age_	14.42	0.00	13	287.48
***ϕ***_sex*age_ **p**_sex*age+habitat_ ***ψ***_**sex+age**_	19.26	0.00	12	294.39

Subscripts include sex (gender), age (second year‐after second year), habitat (campground‐forest), rain (breeding season rainfall), SOI (linear relationship with Southern Oscillation Index [SOI]), SOIsq (quadratic relationship with SOI), density (*t−*1), .male (male response to density in prior year), rep.succ (habitat‐specific reproductive success in prior year).

**Table 3:** Apparent annual survival and habitat‐specific movement rates by age and sex for American redstarts. Values shown are the model‐averaged estimates and 95% confidence intervals from models prior to including annual covariates

Class	Apparent annual survival	Breeding dispersal^a^	*n*
After second year (ASY) female	0.39 (0.26–0.53)	0.03 (0.01–0.14)	117
Second year (SY) female	0.35 (0.20–0.55)	0.09 (0.03–0.28)	94
ASY male	0.33 (0.27–0.39)	0.07 (0.03–0.13)	261
SY male	0.15 (0.09–0.23)	0.18 (0.06–0.40)	184

a Shown for transition from forest to campground.

Movement probabilities between habitats were lowest for ASY females, similar for SY females and ASY males, and highest for SY males (Table 3). A model with sex*age interactions had lower support than a sex+age model, with males showing a higher probability of moving between habitats than females but similar differences with age for each sex. Our models did not support a difference in the direction of movement between habitats although this may have been a sampling limitation. Of 12 male movements, 10 were from forest to campground, while 2 were the reverse. For females, all 3 movements were from campground to forest.

### Objective 2: influence of annual covariates on apparent survival and movement

**Figure 2:**
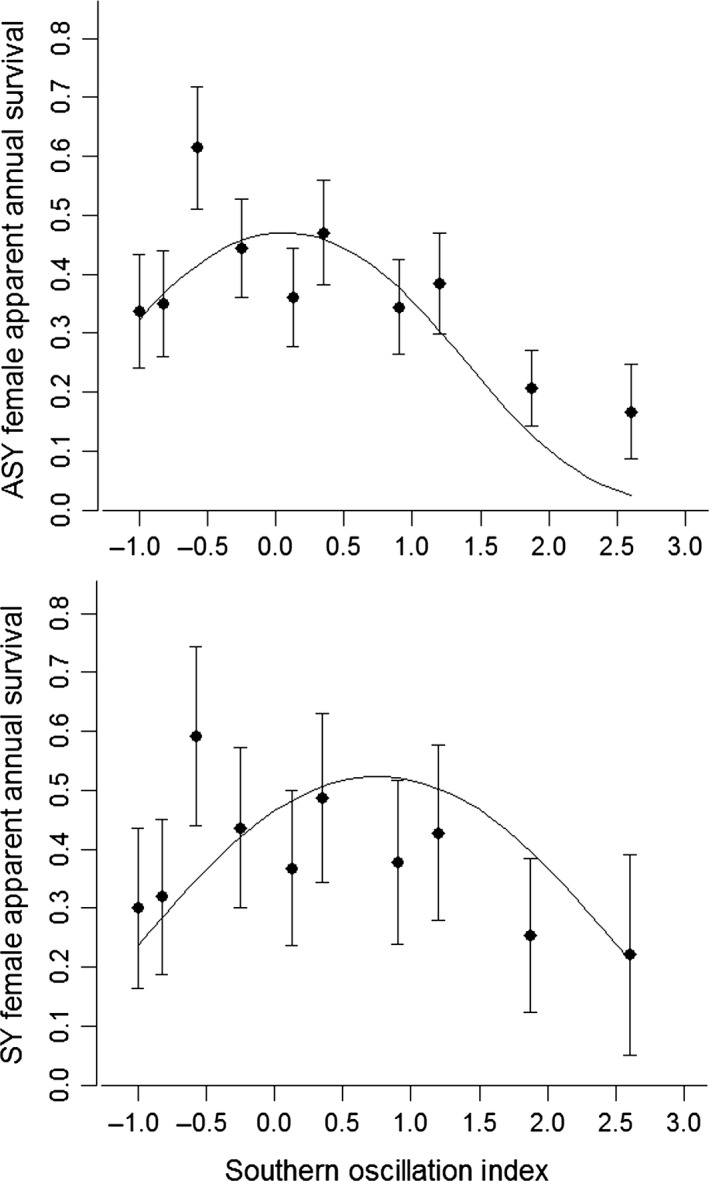
Relationship between the SOI (Southern Oscillation Index) and apparent annual survival of female American redstarts in southeastern Ontario, 2001–2011. Annual survival estimates (mean ± SE) were derived by model averaging across the candidate set and are plotted against the winter SOI. Solid lines are the predicted estimates from the second best model in Table 2 with an SOIsq by age interaction. There was no evidence for different responses of males and females and we only plot results for females to illustrate the relationship.

Apparent annual survival displayed a curvilinear relationship with the SOI and was highest at intermediate values (${\hat{\beta }}_{\mathrm{SOI}}$ = 0.22, [95% CI: *−*0.14, 0.57], $\beta_{\mathrm{SOI}.\mathrm{sq}}$ = *−*0.37, [*−*0.60, *−*0.15], Table 2, Fig. 2). There was no evidence for males and females to differ in this relationship, and while there was some support for a model with a quadratic SOI (SOI_sq_) by age interaction, the predictions from that model showed similar patterns for the two age classes. There was no evidence for an influence of NDVI from Cuba/Jamaica on apparent annual survival as a uniform response (${\hat{\beta }}_{\mathrm{WestNDVI}}$ = 0.03 [*−*0.15, 0.21]) or as an interaction with age or sex. The addition of NDVI from Hispaniola/Puerto Rico led to a drop in QAICc = 3.54 with a negative response of survival to NDVI (${\hat{\beta }}_{\mathrm{eastNDVI}}$ = *−*0.23 [*−*0.43, *−*0.04]). The response was stronger for ASYs (${\hat{\beta }}_{\mathrm{eastNDVI}}\_\mathrm{ASY}$ = *−*0.35, [*−*0.58, *−*0.12]) than SYs (${\hat{\beta }}_{\mathrm{eastNDVI}}\_\mathrm{SY}$ = 0.10, [*−*0.27, 0.47]), but showed no difference by sex.

We found a negative linear relationship between breeding ground rainfall and apparent survival (${\hat{\beta }}_{\mathrm{rain}}$ = *−*0.23, (*−*0.44, *−*0.02), $R_{\mathrm{dev}}^{2}$ = 0.199), with no variation by age or sex. However, this negative effect appeared to be largely due to a single year of high survival from 2002 to 2003 when conditions were unusually dry during the 2002 breeding season (standardized rainfall = *−*2.18). A re‐analysis of the data with rainfall for that year set to the average resulted in a higher QAICc than a model without rainfall. Breeding ground temperature led to a drop in QAICc when included individually (${\hat{\beta }}_{\mathrm{temp}}$ = 0.14, [*−*0.06, 0.34], $R_{\mathrm{dev}}^{2}$ = 0.077, Table 2), but dropped out of top models that included rainfall and SOI, perhaps because wetter years are cooler on average. Addition of breeding season density in year *t − *1 did not improve model support, either as a uniform effect ($\beta_{\mathrm{density},\mathrm{sury}}$ = 0.13, [*−*0.09, 0.36], $R_{\mathrm{dev}}^{2}$ = 0.038) or as an interaction by age or sex (Table 2).

**Figure 3:**
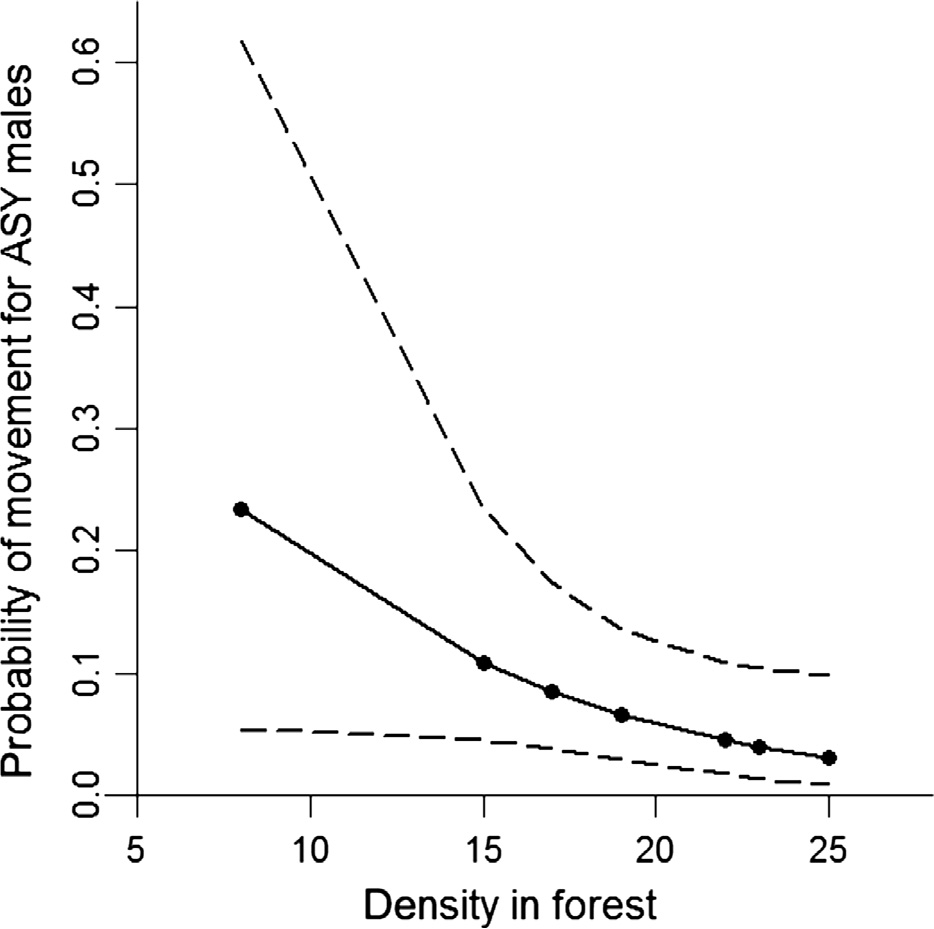
Predicted movement of after second year male American redstarts in relation to habitat‐specific density in southeastern Ontario, 2001–2011. Values are the model‐averaged estimates (±95% CI) and show the predicted movement from forest to campground in relation to the density in a male's habitat from the previous year. Note that seven rather than ten data points are shown due to density in forest being the same for some years.

Male movement between habitats was negatively density dependent: individuals were less likely to move as the density at their former habitat increased ($\beta_{\mathrm{density},\mathrm{male}\mathrm{disp}}$ = *−*0.63, [*−*1.31, 0.03], Fig. 3, Table 2). With both males and females included, the coefficient was still negative but weaker and the confidence intervals overlapped zero to a greater extent (${\hat{\beta }}_{\mathrm{density},\mathrm{disp}}$ = *−*0.33, [*−*0.92, 0.24], Fig. 3, Table 2). There was no evidence that the average fledging success for a habitat in year *t − *1 had an effect on movement between habitats in year *t* (both sexes: $\beta_{\mathrm{success}}$ = *−*0.35, [*−*1.14, 0.45], males only: ${\hat{\beta }}_{\mathrm{success},\mathrm{male}\mathrm{disp}}$ = *−*0.30, [*−*1.16, 0.56], Table 2).

## Discussion

Mark–recapture studies of avian survival typically assess apparent survival, but often less attention is paid to temporally varying factors that could influence dispersal rates. We examined age‐ and sex‐specific apparent survival rates in a population of American redstarts over 11 years. To examine one component of breeding dispersal, we made use of two distinct but adjacent habitat types within our study area to examine movements between habitat types. Finally, we assessed the influence of potential drivers on rates of survival and movement to test various predictions. Apparent annual survival was generally low for all age and sex classes (<0.4), and particularly for SY males (0.15), but this group also showed the highest movement rates between habitat types. We found support for the hypothesis that global climate cycles influence survival. However, while we predicted that survival would be higher during La Niña events, we found lower survival during El Niño and La Niña conditions with higher survival in average years. We found support for the hypothesis that breeding density influences movement rates between contiguous forest and more disturbed campground habitat types, but no evidence that movement was affected by habitat‐specific reproductive success.

### Apparent annual survival and movement rates

Apparent annual survival of American redstarts breeding in Ontario (Table 3) was generally lower than previous estimates from elsewhere across the breeding and nonbreeding range, particularly for SY males. In New Hampshire, USA, and Saskatchewan, Canada, annual survival for all age and sex classes combined was 0.67 and 0.55, respectively (Nichols et al. 1981; Bayne and Hobson 2002). In Jamaica, annual survival ranged from 0.28 to 0.58 across different age and sex classes and habitat types, but was generally >0.4 (Johnson et al. 2006; Marra et al. 2015). We suspect that substantial movement of birds, rather than strictly lower survival, could have played a role in the below‐average survival rates on our study site.

Despite a low overall number of movement events between forest and campground habitat types on our study site, we were able to estimated movement rates that ranged from 0.03 for ASY females to 0.18 for SY males. These values could be interpreted as minimum estimates of true breeding dispersal. In other words, the movement rates that we calculated represent small‐scale local movements (<1400 m; Fig. 1) that are only one component of true breeding dispersal. We were not able to account for long‐distance dispersal events that could have resulted in permanent emigration when birds moved out of our study site. For example, recent work using stable isotope techniques has shown that long‐distance dispersal distances can be substantial (i.e., hundreds of km) and can be influenced by conditions experienced by birds on the nonbreeding grounds – although these dispersal events are most common during natal dispersal (i.e., from hatch year to SY; Studds et al. 2008; Rushing et al. 2015a). Similarly, we could not account for small‐scale movement that could have occurred if birds moved to nearby territories just outside of our study site boundaries, but within the same habitat type. Indeed, we observed a number of small‐scale movements (<500 m) of birds which remained within the same habitat type on our study site from year to year. Interestingly, the semi‐fragmented nature of our study region could have influenced the dispersal behavior of birds. For example, our estimates of apparent survival were most similar to another highly fragmented landscape in central Alberta where survival rates were 0.40 for ASY males and 0.23 for SY males (S. Hannon, unpubl. data). Thus, although we have suggestive evidence for breeding dispersal influencing apparent survival rates, particularly for SY males which showed a strong negative correlation between apparent survival and movement rates, a full consideration of movement at multiple scales would be needed to fully understand this relationship.

Comparison of our results with other studies where movement may be more limited can provide a sense of the proportion of individuals leaving our study area between breeding seasons. Studies on Neotropical migrants have found that the vast majority of annual mortality occurs during the nonbreeding period (Sillett and Holmes 2002; Sillett & Marra, unpubl. data). For American redstarts, individuals that breed in eastern North America are thought to overwinter primarily in the Greater Antilles (Norris et al. 2006). Thus, long‐term field studies of American redstarts in Jamaica likely sample the same overall population as our study population in Ontario, and they likely experience similar sources of mortality throughout the annual cycle. Estimates of apparent annual survival in high‐quality mangrove habitat in Jamaica were 0.50 for SY males and 0.59 for ASY males, and while some interannual movement may occur, it is likely low (Marra et al. 2015). If annual survival is similar between redstarts that breed on our study site and those that overwinter in Jamaica, this would suggest that only 15% of SY breeders in year *t* survive and return to the study area in year *t* + 1, while about twice as many would survive and return, but breed outside of the study area. Of those that did return to the study area, we found that 18% on average switched habitats. If this approach is applied to the other age classes, it suggests that roughly one quarter of ASY males and <10% of females would return but breed elsewhere. We acknowledge that these are only coarse estimates dependent on several assumptions but they provide an approximate extent of breeding dispersal beyond what we were able to measure in this study.

### Influence of annual covariates on apparent survival

Survival of American redstarts was highest at intermediate values of the SOI and declined under La Niña and El Niño conditions. This is in contrast to previous work showing positive effects of an increasing SOI (La Niña conditions) on survival in other Neotropical migrants (Sillett et al. 2000; Mazerolle et al. 2005). It should be noted, however, that no strong El Niño events occurred during the course of our study, in contrast to several strong El Niño events and no strong La Niña events in the 1990s that were reported in previous work (Sillett et al. 2000; Mazerolle et al. 2005). In our study, apparent survival was particularly low during the La Niña event of 2010–2011, the strongest La Niña episode in the past century, when only two of 51 individuals known to be alive in 2010 were observed in the study area. While El Niño events have been associated with lower winter rainfall (Ropelewski and Halpert 1987) which may contribute to reduced body condition of redstarts (Studds and Marra 2007), La Niña events generally result in higher winter rainfall, and thus it is not clear why this would contribute to lower survival. A recent study of a nonbreeding population of American redstarts in Jamaica also reported a negative influence of increasing rainfall on apparent survival (Marra et al. 2015). Taken together, these results suggest that both weather extremes may have negative effects on American redstart survival. While El Niño conditions have been linked to survival through effects on rainfall, perhaps larger scale effects of La Niña conditions influence individuals throughout the annual cycle. For instance, La Niña conditions are associated with tropical storm frequency in the Caribbean (Vitart and Anderson 2001), which could negatively affect individuals during migration or winter. Another intriguing possibility is that a joint influence of the El Niño Southern Oscillation on departure decisions from the wintering grounds and weather on the breeding grounds constrains survival in redstarts. Higher rainfall in Jamaica is associated with earlier departure from Jamaica (Studds and Marra 2011) and earlier arrival in Ontario (McKellar et al. 2013), and birds arriving too early may face unfavorable conditions such as cold weather in April and May that is typical of La Niña events in eastern Canada and the northeastern United States (Shabbar 2006). We did not find strong evidence for an age or sex difference in the influence of the SOI on survival.

Overall, winter NDVI from the larger of the nonbreeding areas (Cuba and Jamaica) did not appear to have any influence on survival, while there was some evidence of a negative effect on survival of NDVI in Hispaniola and Puerto Rico, which was stronger in ASY males. This was contrary to our predictions and may have been driven by correlations between the NDVI and SOI, and the generally more negative association between SOI and survival (Fig. 2).

Density‐dependent population regulation has been observed across a wide range of taxa (Newton 1998; Coulson et al. 2001). In American redstarts, annual survival decreases with increasing density in high‐quality habitat on the Jamaican nonbreeding grounds (Marra et al. 2015), and fecundity decreases with increasing density on the Ontario breeding grounds (McKellar et al. 2014), but we did not observe density‐dependent survival on the breeding grounds. Similarly, density‐dependent fecundity, but not annual survival, was observed in a breeding population of black‐throated blue warblers (*Setophaga caerulescens*) (Sillett and Holmes 2005). The lack of density‐dependent survival on the breeding grounds could be a reflection of generally high oversummer survival of migratory birds (Sillett and Holmes 2002), indicating that negative effects of density such as crowding do not influence annual survival on the breeding grounds. In contrast, on the nonbreeding grounds, crowding results in a decrease in body condition and likely later departure in American redstarts, possibly increasing mortality risk during subsequent phases of the annual cycle (Marra et al. 2015). The contrasting patterns of density dependence in breeding and nonbreeding areas could have implications for management and conservation of migratory populations across the annual cycle (Sheehy et al. 2010).

### Influence of annual covariates on movement

Both positive and negative density‐dependent dispersal have been observed in a variety of bird and mammal species (Matthysen 2005). In a playback experiment, Hahn and Silverman (2006) found evidence for negative density‐dependent dispersal caused by conspecific attraction in American redstarts breeding in Michigan, USA. Our results provide further evidence for conspecific attraction in redstarts, because returning birds were less likely to disperse between habitat types when the density at their previously occupied habitat was greater (Fig. 3). This pattern was strongest in males, although sample sizes were much lower for females. While the results of Hahn and Silverman (2006) were driven primarily by new ASY males recruiting into the population (see also Rushing et al. 2015b), our results demonstrate that returning males may also respond to local density in their settlement decisions. Male redstarts may benefit from settling in dense areas due to increased opportunities for extra‐pair paternity, although they may face a trade‐off due to greater paternity losses in these areas (McKellar et al. 2014). Interestingly, our results and those of Studds et al. (2008) and Rushing et al. (2015a) suggest that different mechanisms may operate at different scales to influence large and small‐scale movement patterns of redstarts (i.e., habitat quality on the nonbreeding grounds vs. density on the breeding grounds), which may have implications for optimal conservation strategies in the face of habitat loss and climate change (Knowlton and Graham 2010).

In contrast to our second dispersal prediction, we found no effect of habitat‐specific fledging success on movement patterns. Previous work has shown that greater numbers of fledglings can attract more adults to that habitat the subsequent season (Doligez et al. 2002; Parejo et al. 2007). We used mean fledging success, which may not have adequately reflected the reproductive output of a habitat. We were not able to use total number of fledglings due to incomplete reproductive data for all pairs. However, recent work with American redstarts breeding in Maryland, USA, also failed to find evidence for the use of public information on breeding habitat selection: experimental plots treated during one breeding season with adult and fledgling calls were no more likely to be settled the subsequent season than were control plots (Rushing et al. 2015b).

## Conclusions

Estimating true survival in open populations is difficult, but new techniques that make use of capture locations to model individual dispersal probabilities are enabling increasingly unbiased estimates of survival (Gilroy et al. 2012; Ergon and Gardner 2014; Schaub and Royle 2014). We used an alternative approach that involved examining dispersal within the study area and can be applied when detailed capture information is not available. One consequence of this approach is that a biologically meaningful dispersal event must be defined by the researcher (Schaub and von Hirschheydt 2009). Here, we defined dispersal events as movements between two distinct habitat types within our study area. This method proved especially useful in our study system because it both aided in the interpretation of unusually low apparent survival and provided insights into proximate causes of breeding dispersal between habitat types. Future work should take into account other forms of dispersal, both large‐scale and small‐scale, in order to adequately distinguish mortality from permanent emigration, which will no doubt be critical to accurately parameterizing population models needed for setting conservation goals (Fletcher et al. 2006).

## Conflict of Interest

None declared.
